# Supplementary material for: Cerebellar Volume Measures Differentiate Multiple Sclerosis Fallers from Non-Fallers
Source: Brain Sci. 2025 Jan 16;15(1):77. doi: 10.3390/brainsci15010077 (PMC11764211; doi:10.3390/brainsci15010077)
Supplement: Supplementary file 1 [file brainsci-15-00077-s001.zip › brainsci-3389969-supplementary.pdf]

Supplementary Table 1. *Comparison of Cerebellar Volumes among MS fallers, MS non-fallers, and HCs*

	**MS (n=31)**	**MS Faller (n=15)**	**MS Non-Faller (n=16)**	**HC (n=29)**
**Corpus Medullare**	11980.81* (1055.54)	11564.73*† (1106.28)	12370.88 (866.40)	12649.83 (1516.50)
**Lobules I-III**	1137.55* (323.50)	1657.13* (262.52)	1812.94 (363.95)	1901.79 (377.42)
**Lobule IV**	6125.71 (705.63)	6063.27 (850.74)	6184.25 (559.26)	6131.24 (837.50)
**Lobule V**	6310.26* (999.08)	6136.33* (988.50)	6473.31 (1012.78)	7043.07 (1019.20)
**Lobule VI**	16830.68 (2498.76)	15941.73*† (2227.01)	17664.06 (2515.48)	17639.34 (2502.42)
**Crus I**	24327.58 (3712.18)	23280.07* (3099.90)	25309.63 (4057.81)	25311.72 (2928.88)
**Crus II**	15100.10 (1909.33)	14843.47 (1500.53)	15340.69 (2249.89)	15304.00 (2480.83)
**Lobule VIIB**	10287.26 (1486.58)	10086.07 (1163.91)	10475.88 (1754.14)	10775.59 (1612.21)
**Lobule VIIIA**	9897.97 (2386.80)	9471.07 (1924.60)	10298.19 (2753.79)	9011.38 (1706.95)
**Lobule VIIIB**	6137.87 (861.57)	6250.13 (853.04)	6032.63 (883.75)	6471.41 (942.73)
**Lobule IX**	5677.81 (915.02)	5735.07 (1095.31)	5624.13 (740.58)	6166.03 (1166.55)
**Lobule X**	889.58 (158.15)	894.07 (156.82)	885.38 (164.41)	899.69 (164.73)
**Vermis VI**	1421.16 (244.70)	1403.93 (238.77)	1437.31 (256.85)	1417.97 (146.04)
**Vermis VII**	970.74 (194.20)	976.80 (151.02)	965.06 (232.53)	1038.69 (185.15)
**Vermis VIII**	1828.03 (300.18)	1765.33 (188.99)	1886.81 (373.18)	1867.34 (358.95)
**Vermis IX**	965.61 (131.83)	913.67† (131.42)	1014.31 (115.81)	993.86 (191.70)
**Vermis X**	319.39 (50.53)	299.00*† (52.38)	338.50 (41.71)	340.62 (66.58)
**Motor Lobules (I-V, VIII)**	30209.4 (3318.3)	29577.9 (2509.2)	30801.3 (3920.9)	30558.9 (2905.2)
**Cognitive Lobules (VI, VII, Crus I-II)**	66545.6 (6382.9)	64151.3*† (5202.9)	68790.3 (6716.5)	69030.7 (6748.5)

All volumes are in mm^3 and listed as mean (SD). * Indicates significantly different from HC at $p < 0.05$. † Indicates significantly different from non-fallers (see Table 2 for p-values).
